# Barriers and Enablers to Total Contact Casting Among Patients With Diabetic Foot Ulcers in Singapore: A Qualitative Study

**DOI:** 10.1002/jfa2.70243

**Published:** 2026-11-14

**Authors:** Jia En Jolene Tai, Kavita Venkataraman

**Affiliations:** ^1^ Saw Swee Hock School of Public Health National University of Singapore and National University Health System Singapore; ^2^ Podiatry Department Ng Teng Fong General Hospital Singapore; ^3^ Yong Loo Lin School of Medicine National University of Singapore and National University Health System Singapore

**Keywords:** barriers, diabetic foot ulcer, enablers, offload, patient perspectives, South‐East Asia, total contact cast

## Abstract

**Introduction:**

Non‐removable knee‐high offloading devices, including total contact casts, are considered the gold standard for offloading diabetic foot ulcers, yet their global uptake remain low. This study aims to explore the barriers and enablers influencing patients’ choice to undergo total contact casting for their diabetic foot ulcers in Singapore’s multicultural urban setting.

**Methods:**

Semi‐structured face‐to‐face interviews were conducted with 11 podiatry patients (7 total contact cast users, 4 decliners) in a Singapore public hospital. A combination of purposive and convenience sampling was used to select patients based on the inclusion and exclusion criteria. Thematic analysis was used to generate new and existing themes.

**Results:**

Patient and intervention related factors were the two key themes. The most frequently reported barriers were impact on mobility and daily function (*n* = 11/11) and low perceived severity of condition (*n* = 4/11). Other barriers to total contact casting include job related concerns, social stigma, psychological fear, limited social support, gait changes, cast complications, therapy expectations, and reduced wound monitoring. Trust in clinicians’ expertise and perceived benefits of total contact cast were key enablers of total contact casting.

**Conclusion:**

This study revealed that resistance to total contact casting reflects a mismatch between evidence‐based clinical recommendations and patients’ lived realities in tropical urban settings, shaped by socioeconomic circumstances. These findings highlight the importance of addressing patients’ needs through targeted education, shared decision making and adequate support. Healthcare policies could consider facilitating access to such support to reduce treatment barriers. Future qualitative studies should expand to explore healthcare professionals’ experiences and incorporate diverse linguistic and demographic groups, given Singapore’s multicultural and multi‐religious landscape. Subsequent mixed‐method studies could examine how socioeconomic factors influence total contact casting uptake and evaluate interventions to improve treatment acceptance.

## 1 Introduction

Singapore’s prevalence of diabetes mellitus is forecast to reach 15% in 2050 from 11.3% in 2010 [1], with up to 34% of individuals with diabetes developing a diabetic foot ulcer (DFU) in their lifetime [2]. Untreated DFUs can result in soft tissue infections, gangrene and lower limb amputations [3]‐with a 5‐year mortality rate of 30% for those with DFUs and mortality rate more than 70% for those with above foot amputation [3]. Alarmingly, Singapore faces one of the highest rates of lower limb amputations [4], creating substantial clinical and economic burdens and reducing patient quality of life [5].

Studies have proven that an increased plantar pressure of the foot is a contributing factor to the development of neuropathic DFUs and is a harbinger of lower extremity amputations [6, 7]. Non‐adherence to appropriate footwear increases ulceration risk [8]. Hence, offloading mechanical tissue stress is regarded as a cornerstone in the management of neuropathic DFUs [9].

The 2023 International Working Group on the Diabetic Foot (IWGDF) guidelines recommend a non‐removable knee‐high offloading device as the first‐choice treatment for a neuropathic plantar forefoot or midfoot ulcer [10]. Depending on individual patient circumstances and available resources, either a total contact cast (TCC) or a non‐removable knee‐high walker boot may be used. They are effective in offloading DFUs due to plantar pressure redistribution, transferring 30% of the plantar load to the cast wall [9]. TCC is also reportedly more cost‐efficient than removable cast walkers or therapeutic shoes ($1136.92 per patient vs. $1628.98 and $1933.75) [11]. This could have resulted in cost savings as DFUs heal within a shorter time frame [12].

Despite the IWGDF 2023 guidelines recommending non‐removable offloading devices such as TCC as the standard [10], clinician uptake remains low at less than 2% [13]. Patient resistance is cited as a primary barrier. The extremely low patient response rate for TCC uptake highlights a significant concern as there exists a disparity between evidence‐based standard of care and the clinical picture in a hospital setting. Although barriers to offloading have been explored previously, qualitative evidence examining patients’ perceptions and decision‐making specifically regarding TCC remains limited, particularly in South‐East Asia [14]. To our knowledge, no previous qualitative study has examined patients’ experiences of TCC within the Singapore healthcare context, where a tropical urbanised environment and high burden of diabetes‐related lower‐limb amputation may shape treatment acceptability in ways unique to Singapore [4]. Thus, this study aimed to identify barriers and enablers to TCC adoption among diabetic patients in Singapore by exploring how local context influences treatment decisions.

## 2 Methods

Ethical approval was obtained from the National Healthcare Group Domain Specific Review Board (DSRB), reference number 2024‐3648.

### 2.1 Study Design

This was an exploratory qualitative study using semi‐structured interviews, analysed using a combined deductive–inductive reflexive thematic analysis. The Consolidated Criteria for Reporting Qualitative Research [15] (COREQ) (Supporting Information S1: COREQ Checklist) was used as a reporting framework for this study.

### 2.2 Sampling and Inclusion Criteria

Patients were selected from the podiatry outpatient clinic of a public hospital in Singapore using purposive and convenience sampling based on the inclusion and exclusion criteria. As this was a qualitative study, no formal statistical sample size calculation was undertaken. A provisional recruitment range of 8–30 participants was established based on existing published methodological recommendations for qualitative interview studies [16]. Recruitment continued until data saturation was achieved. Patients were eligible if they were aged 21 years or older, restricting recruitment to adults in accordance with the definition of adulthood under Singapore’s Human Biomedical Research Act. Patients were included if they had a history of type 1 or 2 diabetes mellitus and a current or history of neuropathic foot ulcer which warrants consideration of TCC. Patients were excluded if they had significant peripheral vascular disease that was considered a contraindication to TCC by the treating podiatrist, not fluent in English, acute infection, high falls risk, cognitive or mental impairment, poor self‐care or pregnant.

### 2.3 Patient Recruitment

Patients were recruited from 1 August 2024 to 1 August 2025. Preliminary screening and recruitment of eligible patients were conducted by the attending podiatrists in the outpatient clinic. Informed written consent was obtained if the eligible patient agreed to participate. Recruitment of participants stopped once data saturation was deemed to be achieved. In total, 13 participants were approached and consented, but 2 dropped out prior to the interview for personal reasons, resulting in 11 completed interviews.

### 2.4 Data Collection

The interviews took place between 1 October 2024 to 31 December 2024, in a private consult room, with only the interviewer and participant present. The interviews were conducted in English and audio‐recorded by the principal investigator who had prior knowledge of conducting in‐depth interviews for qualitative research. Face‐to‐face interviews were conducted using a semi‐structured topic guide (Supporting Information S1: Topic Guide), with sessions ranging from 10 to 30 minutes. The preliminary topic guide was founded on the existing knowledge and clinical experience of the study team and findings from the literature review. Demographic data (Table 1) were collected to enhance interpretability of the data collected. Participants were selected to ensure adequate representation, including those who underwent TCC and those who declined it.

**TABLE 1:** Characteristics of participants with DFUs or Charcot foot who participated in individual interviews (*N* = 11).

No.	Age	Gender	Ethnicity	Occupation	Indication for TCC	Duration of wound	Current choice of offloading modality	Done TCC before?
P01	64	Male	Chinese	Brewery factory supervisor	Diabetic neuropathic ulcer	2 years	Diabetic footwear with total contact insoles	Yes
P02	54	Male	Chinese	Part time school warden	Diabetic neuropathic ulcer	5 years	Interchanges between aircast boot and covered footwear with total contact insoles	Yes
P03	41	Male	Chinese	Grab driver	Diabetic neuropathic ulcer	4 years	Sports shoes with total contact insoles	No
P04	47	Male	Indian	Unemployed	Diabetic neuropathic ulcer	6 years	Diabetic footwear with chairside insoles, wound sandal	Yes
P05	48	Male	Malay	Unemployed	Diabetic neuropathic ulcer	5 years	Diabetic footwear with total contact insoles, wound sandal, aircast boot	No
P06	54	Male	Indian	Unemployed	Diabetic neuropathic ulcer	5 years	Diabetic footwear, wound sandal	Yes
P07	63	Female	Indian	Bus attendant	Diabetic neuropathic ulcer and charcot	5 years	Diabetic footwear with total contact insoles, aircast boot	Yes
P08	52	Male	Chinese	Part time cashier	Diabetic neuropathic ulcer	5 years	Sport shoes with total contact insoles, wound sandal	No
P09	54	Male	Chinese	School canteen shop owner	Diabetic neuropathic ulcer	7 years	Diabetic footwear with total contact insoles, wound sandal	No
P10	58	Female	Chinese	Unemployed	Charcot arthropathy	9 years	Sports shoes with total contact insoles	Yes
P11	56	Male	Malay	Unemployed	Diabetic neuropathic ulcer and charcot arthropathy	7 years	Diabetic footwear with total contact insoles	Yes

Interviews were audio‐recorded using two mobile phones, with one serving as a backup. Both devices were stored in a locked hospital clinic drawer when not in use. Recordings were transferred to password‐protected storage accessible only to the principal investigator and subsequently deleted from both devices. Personal identifiers were removed, and transcripts were anonymised using participant pseudonyms. Participants were informed that they could pause or withdraw at any time without affecting their clinical care.

### 2.5 Data Analysis

The recordings were manually transcribed verbatim by the principal investigator. After which the principal investigator reviewed and corrected each transcript against the original audio recording to ensure accuracy. Transcripts were uploaded onto Atlas.ti, a qualitative data analysis software. Initial coding and theme development were undertaken by the principal investigator. Themes were considered in relation to those previously described in the literature while allowing context‐specific themes to be developed from the present data, providing sensitising concepts. Possible themes were subsequently discussed with a study team member for critical reflection and refinement of interpretation. There was no patient involvement in this research.

## 3 Results

Of the 11 participants interviewed, 7 had previously undergone TCC, while 4 had declined the treatment (Table 1).

The findings have been classified under two themes—patient and intervention related factors. Direct quotes have been incorporated to expand on the themes (Supporting Information S1: Additional Quotes).

### 3.1 Barriers

#### 3.1.1 Theme 1—Patient Related Factors

##### 3.1.1.1 Impact on Mobility and Daily Functioning

A prominent theme that emerged from this study concerned the impact of the cast on participants’ daily activities and mobility. Many participants described the use of TCC as highly inconvenient, primarily due to the associated physical discomfort, especially in Singapore’s tropical and humid climate. Some reported experiencing itchiness and sweating after wearing the cast for prolonged durations.

It’s a bit inconvenient when I’m taking shower…When I wet that, you might have some more infections.

(P10, 58‐year‐old female, unemployed)

One participant recounted experiencing additional financial strain, as the cast’s bulk necessitated reliance on taxis or private‐hire services instead of public transport.

Like any other place, you want to go for a wedding. Cannot. Because too heavy, you have to carry. You go anywhere also, you have to take taxi.

(P06, 54‐year‐old male, unemployed)

##### 3.1.1.2 Perceived Low Severity

This group of participants consisted of males in their mid‐forties and fifties who were physically able and employed. Participants understated the urgency of their condition, viewing it as insufficiently severe to warrant TCC and preferring to ‘wait it out’ until necessary. They have a low perceived susceptibility to infection and/or amputation as they believe they are in control of their current wound situation and have taken active self‐care measures.

I don’t think my wound is so serious enough that a cast is needed… So, I do my part… diligently come for your appointment… And then, seek immediate attention once something is wrong…

(P08, 52‐year‐old male, self‐employed)

One participant revealed a knowledge gap, misunderstanding TCC’s role in DFU management and associating it primarily with orthopaedic fractures.

Cast like mainly what for… uh bone fractures or bone crack… something like sports injury… but what about this? I don’t know…

(P03, 41‐year‐old male, self‐employed)

##### 3.1.1.3 Psychological Fear From Cast

Psychological fear precipitated from the cast could also hinder TCC usage. One participant described feelings of helplessness and frustration when unable to remove the cast to relieve leg cramps, ultimately developing what he described as ‘cast phobia.’

Suddenly there’s one cramp… I can’t do much… I’ll be feeling very pain and upset lah… I feel a bit worried when things come back again… I don’t know how to resolve this problem.

(P02, 54‐year‐old male, self‐employed)

##### 3.1.1.4 Job Related Concerns

Employment‐related concerns were barriers to TCC acceptance, particularly among participants whose jobs required prolonged standing or walking. A self‐employed private‐hire driver reported that TCC would prevent him from driving, affecting his livelihood. Another participant working part‐time on a contract basis expressed concerns about prolonged treatment disrupting employment. These findings highlight how occupational demands and financial insecurity may influence TCC acceptance.

I’m a driver… I put the cast, how do I accelerate my pedal and brake… cannot mah, so it affects my job.

(P03, 41‐year‐old male, self‐employed)

##### 3.1.1.5 Lack of Social Support

The absence of social support also influenced decisions to decline TCC, particularly among those living alone or elderly individuals without caregiving assistance. Participants expressed concerns about managing daily activities independently, citing safety and fall risk as challenges.

But that one, for me, is difficult for me… I would say not safe for me because I’m alone at home to handle everything.

(P05, 48‐year‐old male, unemployed)

##### 3.1.1.6 Social Stigma

One participant who rejected the TCC and/or any form of removable cast walkers highlighted the stigma accompanied by the cast – people unfamiliar with medical knowledge might associate it with something serious. These participants might resort to concealing their underlying diabetic foot condition to avoid stigma as it could have a potential impact on their social, work and personal relationships.

The boss at that company told me they don’t want to employ me because they saw me wearing the boot…

(P05, 48‐year‐old male, unemployed)

#### 3.1.2 Theme 2—Intervention Related Factors

##### 3.1.2.1 Reduced Means to Monitor Wounds

As dressing changes could only be performed by the specialist every three to four days, several participants expressed unease about not being able to independently inspect their wounds. This lack of control over wound observation was described as a source of anxiety and distress.

Yeah… that’s why I used to every 2 or 3 days will take a look at my wound but now totally no chances. So the worry will be there. Sometimes I don’t know how the wound progressing…

(P02, 54‐year‐old male, self‐employed)

##### 3.1.2.2 Changes in Gait Patterns

Participants reported that the cast’s rigidity altered their gait and made walking difficult. Many described the adjustment as physically exhausting, citing the cast’s weight and inflexibility as compounding challenges.

Like something in the way when I want to walk. Like cannot walk properly.

(P04, 47‐year‐old male, unemployed)

##### 3.1.2.3 Cast Therapy Expectations

Given that complete healing of a DFU typically requires 4–12 weeks of TCC, some participants expressed reluctance to commit to the therapy, particularly when it posed potential disruptions to their work and they were unable to afford prolonged time away from employment.

Personally, I’m working part‐time and on a contract basis… And work requires me to move around a bit. So being in a cast, even though with a letter from the hospital, it’s not going to help me a lot.

(P08, 52‐year‐old male, self‐employed)

##### 3.1.2.4 Cast Complications

Concerns about potential cast‐related complications, such as iatrogenic skin lesions, discouraged some participants from trying or continuing TCC. Those with prior negative experiences were particularly apprehensive about recurrence.

The restriction there they will rub when my movement is big, the ankle point… or the surface of my skin will rub against the cast.

(P02, 54‐year‐old male, self‐employed)

### 3.2 Enablers

#### 3.2.1 Theme 1—Patient Related Factors

##### 3.2.1.1 Trust in Clinicians’ Expertise

Participants expressed trust in clinicians to recommend treatments that would be beneficial and cause no harm. As TCC application requires specialised skills and is highly clinician‐dependent, participants indicated a greater willingness to adopt the therapy when they had confidence in the clinician’s competency and judgement.

I mean… they are professionals… You have to trust them… they are more knowledgeable than the patients honestly.

(P02, 54‐year‐old male, self‐employed)

#### 3.2.2 Theme 2—Intervention Related Factors

##### 3.2.2.1 Benefits of TCC

Despite TCC limitations, some participants recognised that the benefits outweigh the costs such as accelerated wound healing and effective pressure offloading. Some also noted improved comfort or pain relief, which contributed to a better quality of life.

I think putting it help to heal the wound faster lah and release the pressure… I don’t feel any pain.

(P01, 64‐year‐old male, employed)

## 4 Discussion

### 4.1 Key Findings and Recommendations

This qualitative study provides the first comprehensive exploration of TCC perceptions within Singapore’s unique urban, multicultural healthcare context. By gathering participants' lived experiences of TCC, this study provides valuable insights that are often overlooked by clinicians. Our findings confirm previously identified barriers [17] while revealing context‐specific factors unique to Singapore’s urban healthcare environment that substantiate why patients decline TCC despite knowing its benefits [13, 18].

Our study identified inconvenience, physical discomfort and restriction to activities of daily living as key barriers to using TCC. Singapore’s year‐round tropical climate creates unique comfort challenges with TCC materials trapping heat and moisture, risking skin maceration that compromises diabetic wound healing. A graduated approach may be considered, with removable cast walkers considered for patients unable to tolerate TCC, as comparable healing outcomes have been reported with good adherence [19, 20].

Some studies have analysed how the presence of a DFU and the use of an offloading device significantly restrict mobility and can result in social isolation [21, 22, 23, 24], which was also highlighted in this study. The participants consistently reported that TCC rendered buses largely inaccessible due to the difficulty navigating high bus steps with the rigid cast, forcing taxi reliance and significantly increasing daily transport costs during the therapy period. This transport accessibility challenge represents a context‐specific barrier not commonly addressed in existing TCC literature, highlighting how urban infrastructure design can inadvertently create additional treatment barriers for patients in high‐density, public transport‐dependent cities. This also highlights how urban mobility and socioeconomic constraints can affect treatment acceptance, underscoring the need to consider transport, occupational and caregiving needs in shared decision‐making.

Our findings align with existing literature showing that patients with diabetic foot ulcerations experience behavioural changes, restrictions, and emotional distress [21, 22, 23, 24]. Our participants also experienced fear and worry due to the inability to remove cast independently, a challenge highlighted in another study as well [24]. Participants’ reports of inadequate social support echo other studies where insufficient family or caregiver assistance leads to poorer outcomes [25, 26]. Potential strategies include engaging trained volunteers to provide check‐ins and practical support during TCC treatment might help reduce fears of isolation and helplessness.

Participants identified social stigma as a key barrier, fearing that TCC would reduce employability and signal illness severity—concerns congruous with other studies where patients dismissed prescribed offloading modalities due to embarrassment and stress about their visible appearance [27]. This stigma‐related barrier suggests the need for culturally sensitive adaptation strategies, including workplace accommodations.

Notably, our participants demonstrated low perceived severity of their wound condition, reinforcing misconceptions found in other studies where patients view offloading modalities—such as TCC or removable cast walkers—as non‐essential for diabetic foot treatment [28], adopting only regular wound dressings [29]. This dissonance between clinical severity and patient perception represents a critical barrier to treatment acceptance with underlying health literacy challenges. These findings highlight the importance of public health initiatives for patient education on the progressive nature of diabetic foot complications and the importance of offloading.

Singapore’s multi‐tiered subsidy system [29] creates access barriers through means‐testing that produces a ‘coverage gap’—middle‐income patients often don’t qualify for higher subsidies yet still face financially burdensome TCC costs. Patients with poorer health literacy can potentially delay treatment initiation due to uncertainty in available funding. This corroborates with studies that showed that some patients declined offloading interventions due to high cost and lack of financial assistance [25, 26, 30, 31]. These findings indicate the need to examine healthcare financing gaps affecting patients who remain financially burdened despite being ineligible for higher subsidies.

Some participants also rejected performing the cast as the expected therapy commitment of 4–12 weeks poses difficulty, especially for blue or grey‐collar working adults. This substantiates other studies whereby patients had to miss work due to the presence of offloading modality, impeding their work and hence impacting their long‐term job security [24]. The physical demands and mobility requirements of their job make TCC particularly challenging for this patient population who may lack workplace flexibility. The possibility of cast complications can also dissuade participants from TCC, and this is consistent in other studies whereby TCC application is clinician dependent and requires skilled expertise. Standardised TCC training, including safe application and regular competency assessments, could boost treatment consistency, patient confidence and clinical outcomes [32, 33].

Trust in clinicians’ expertise plays a crucial role in patients’ willingness to undergo TCC. This aligns with findings from other studies emphasising the importance of building strong patient‐clinician relationships and rapport to enhance adherence to offloading interventions [27, 31]. However, while many participants demonstrated sufficient understanding of TCC benefits, they often struggled to fully grasp the severity of their wound condition. Participants tended to prioritise immediate, tangible concerns—such as maintaining their livelihood—while failing to recognise long‐term consequences, including amputation risk, if the condition remains improperly managed. This temporal disconnect between short‐term inconvenience and long‐term outcomes limits TCC acceptance. Setting clear expectations regarding cast requirements and outcomes, supported by peer testimonials, may aid informed treatment decisions and patient understanding.

### 4.2 Strengths, Limitations and Reflexivity

To our knowledge, this is the first qualitative study exploring barriers and facilitators to TCC for DFUs from the patient perspective in both South‐East Asia and Singapore, addressing a significant gap in regional literature.

This study provided valuable initial insights into TCC experiences in Singapore’s context, while acknowledging several areas of limitation that influenced the representativeness and transferability of findings. The sample size (*n* = 11) achieved data saturation and yielded rich, in‐depth narratives that illuminate important patient perspectives previously unexplored in the South‐East Asian context, despite having a sampling bias that limited its generalisability. The study population was predominantly male (9/11 participants), recruited from a single institution, and restricted to English‐speaking participants. The study’s focus on English‐speaking participants provided clear, detailed accounts of patient experiences, though future research would benefit from multilingual approaches to capture Singapore’s diverse cultural perspectives, including insights from Malay, Tamil, and Chinese‐speaking communities. Our study did not manage to explore potential religious influences on TCC acceptance, which will be relevant given Singapore’s diverse religious landscape and the potential impact of religious practices on treatment adherence. While barriers were substantially more numerous than enablers (10 vs. 2 themes), this reflects both the genuine challenges patients face and potential sampling effects—decliners may have more articulated reasons for refusal than accepters have for acceptance. Future research should specifically probe factors that sustain TCC adherence among successful users.

The gender composition may have influenced our thematic findings, as our study’s male predominance likely amplified employment and occupational concerns as primary barriers. Female participants may prioritise different factors such as family caregiving and aesthetics. Future studies should include more diverse gender representation to better capture differences in TCC experiences.

The principal investigator’s dual role as both an experienced DFU podiatrist and a graduate student conducting qualitative research presented both methodological considerations and advantages. This dual positionality provided valuable clinical background knowledge, enabling informed questioning and deeper engagement with participants’ narratives [34]. However, it may have influenced participant responses through social desirability bias [35], with participants potentially reluctant to criticise treatment recommendations or express negative experiences to their healthcare provider. To address these considerations, strict confidentiality protocols were maintained and clear researcher‐participant boundaries were established [36]. A reflexive approach was adopted, and the investigator was mindful of power dynamics during interviews, ensuring that participants felt comfortable sharing their experiences without undue influence from clinical perspectives. Collaborative coding by the principal investigator and study team member enhanced trustworthiness. Future studies could strengthen this further with independent coding by multiple researchers and member checking.

## 5 Conclusion

This study revealed that resistance to TCC reflects a mismatch between evidence‐based clinical recommendations and patients’ lived realities in tropical urban settings, shaped by socioeconomic circumstances. These findings highlight the importance of addressing patients’ needs through targeted education, shared decision making and adequate support. Healthcare policies should consider facilitating access to such support to reduce treatment barriers. Future studies should expand to explore healthcare professionals’ experiences and incorporate diverse linguistic and demographic groups, given Singapore’s multicultural and multi‐religious landscape. Subsequent mixed‐method studies could examine how socioeconomic factors influence TCC uptake and evaluate interventions to improve treatment acceptance.

## Author Contributions

**Jia En Jolene Tai:** conceptualisation, methodology, investigation, data curation, formal analysis, writing – original draft. **Kavita Venkataraman:** methodology, supervision, writing – review and editing.

## Funding

The authors have nothing to report.

## Conflicts of Interest

The authors declare no conflicts of interest.

## Supporting information


Supporting Information S1

## Data Availability

The interview transcripts generated during this study are not publicly available due to participant confidentiality and ethical restrictions. Relevant anonymised excerpts supporting the findings are provided within the article and its Supporting Information.
