# Supplementary material for: Barriers and Enablers to Total Contact Casting Among Patients With Diabetic Foot Ulcers in Singapore: A Qualitative Study
Source: J Foot Ankle Res. 2026 Nov 14;19(4):e70243. doi: 10.1002/jfa2.70243 (PMC13769385; doi:10.1002/jfa2.70243)
Supplement: Supplementary file 1 [file JFA2-19-e70243-s001.docx]

**Supplementary Information S1: COREQ Checklist**

*The Consolidated Criteria for Reporting Qualitative Studies (COREQ): 32-item checklist*

| **No. Item** | **Guide questions/description** | **Notes** |
| --- | --- | --- |
| Domain 1: Research team and reflexivity | | |
| The research team had expertise in diabetes (JT, KV), diabetic foot conditions (JT), qualitative methodology (KV) and public health (KV). | | |
| Personal Characteristics | | |
| 1. Interviewer/facilitator | Which author/s conducted the interview or focus group | JT conducted all semi structured interviews. |
| 2. Credentials | What were the researcher’s credentials? E.g. PhD, MD | JT: Bachelor of Health Science and Master in Podiatric Practice  KV: MBBS, PG DHA, PHD |
| 3. Occupation | What was their occupation at the time of the study? | JT: Podiatrist and post graduate student  KV: Associate Professor |
| 4. Gender | Was the researcher male or female? | Both were females |
| 5. Experience and training | What experience or training did the researcher(s) have? | JT had undergone a module in qualitative methods. KV had experience in qualitative methods, including publishing qualitative study papers. |
| Relationship with participants | | |
| 6. Relationship established | Was a relationship established prior to study commencement? | JT had an ongoing professional relationship with the participants as she is a podiatrist attached to the hospital of the recruited participants prior to obtaining informed consent. However, informed consent was obtained by another treating podiatrist to reduce coercion. KV has no prior relationship with the participants as she does not work in that hospital. |
| 7. Participant knowledge of the interview | What did the participants know about the researcher? e.g. personal goals, reasons for doing the research | Participants were aware that this was a research project to explore the barriers and enablers towards total contact casting in diabetic foot ulcers and/or Charcot. The research team explained that the goal of the research was to better guide and tailor future interventions to improve overall patient outcomes and reduce long term diabetic foot amputations. |
| 8. Interviewer characteristics | What characteristics were reported about the inter viewer/facilitator? e.g. Bias, assumptions, reasons and interests in the research topic | The research team had an interest in diabetic foot and were invested in understanding offloading interventions for patients with diabetic foot ulcers. |
| Domain 2: Study design | | |
| Theoretical framework | | |
| 9. Methodological orientation and Theory | What methodological orientation was stated to underpin the study? e.g. grounded theory, discourse analysis, ethnography, phenomenology, content analysis | The research team conducted thematic analysis. We analysed the data in an inductive manner and matching themes in current literature while coming up with new themes. |
| 10. Sampling | How were participants selected? e.g. purposive, convenience, consecutive, snowball | We used a combination of purposive and convenience sampling to attempt to reach a diverse range of participants |
| 11. Method of approach | How were participants approached? e.g. face-to-face, telephone, mail, email | We recruited all patients from the Podiatry outpatient clinic face to face during their podiatry reviews with the attending clinician. |
| 12. Sample size | How many participants were in the study? | 11 participants |
| 13. Non-participation | How many people refused to participate or dropped out? Reasons? | Out of the 13 participants recruited, 2 dropped out before the interview due to personal reasons. |
| Setting | | |
| 14. Setting of data collection | Where was the data collected? e.g. home, clinic, workplace | Interview sessions were conducted face to face in a private consult room |
| 15. Presence of nonparticipants | Was anyone else present besides the participants and researchers? | There were no people present during the data collection besides participants and the principal researcher. |
| 16. Description of sample | What are the important characteristics of the sample? e.g. demographic data, date | All participants have type 2 Diabetes and history of neuropathic foot ulcers and/or Charcot foot. The average age of the participants was 53. Out of all the participants, only 2 were females. |
| Data collection | | |
| 17. Interview guide | Were questions, prompts, guides provided by the authors? Was it pilot tested? | See section “Data collection”, page 7 |
| 18. Repeat interviews | Were repeat interviews carried out? If yes, how many? | There were no repeat interviews with the same participants. |
| 19. Audio/visual recording | Did the research use audio or visual recording to collect the data? | All interviews were audio recorded with participants’ permission. |
| 20. Field notes | Were field notes made during and/or after the interview or focus group? | The principal researcher made field notes during the interviews. |
| 21. Duration | What was the duration of the interviews or focus group? | The interviews with participants ranged from 10 to 20 minutes. Average time was 12 minutes. |
| 22. Data saturation | Was data saturation discussed? | Data saturation was deemed to be achieved as the major themes were all covered. |
| 23. Transcripts returned | Were transcripts returned to participants for comment and/or correction? | Transcripts were not returned to participants for comment and/or correction as all participants declined to review them. |
| Domain 3: Analysis and findings | | |
| Data analysis | | |
| 24. Number of data coders | How many data coders coded the data? | The primary researcher coded the interviews individually and developed a preliminary coding tree. The primary researcher used this coding tree to gather feedback with the other team member (who is also her practicum supervisor) and refined the codes accordingly. |
| 25. Description of the coding tree | Did authors provide a description of the coding tree? | There is no description of the coding tree. |
| 26. Derivation of themes | Were themes identified in advance or derived from the data? | Themes were derived from the data in an inductive manner and those current themes were matched back to existing literature, while new themes emerged. |
| 27. Software | What software, if applicable, was used to manage the data? | The primary researcher used Atlas.ti for the coding of all interviews. |
| 28. Participant checking | Did participants provide feedback on the findings? | The participants did not provide any feedback nor contributed to the findings. |
| Reporting | | |
| 29. Quotations presented | Were participant quotations presented to illustrate the themes/ findings? Was each quotation identified? E.g. participant number | Key findings of this study were supported with selected quotes in text. Additional supporting quotes are provided in Supplementary 3. |
| 30. Data and findings consistent | Was there consistency between the data presented and the findings? | All findings were derived from the data and all themes are supported by illustrative quotes. |
| 31. Clarity of major themes | Were major themes clearly presented in the findings? | Major themes were derived from the data and are clearly defined by a paragraph title. |
| 32. Clarity of minor themes | Is there a description of diverse cases or discussion of minor themes? | Themes on which there was a varying opinion within the study team are discussed. Themes that are only described by one participant are also discussed in the results section of this study. |

**Supplementary Information S2: Topic Guide**

| Good morning/afternoon Mr****/Mdm****. Thank you for taking the time to participate in this study. As mentioned in the consent form previously, I’ll be conducting an in -depth interview with you regarding your diabetic foot and perceptions behind total contact casting. I would like to ask you some questions related to your health, particularly your diabetic foot problem and footwear modality i.e. total contact cast. This interview will be audio-recorded as we do not want to miss any important points. Please be assured that this conversation will remain anonymous, confidential and only myself and 2 of my colleagues will have access to the audio recordings and the transcripts. There will be no names associated with any comments in my final report. Thank you | |
| --- | --- |
| **Theme** | **Suggested Questions** |
| Demographics | [Pre-Interview Survey]   - Age - Gender - Ethnicity - Religion - Occupation - Indication for TCC - Duration and location of wound - Choice of offloading modality (TCC vs others) |
| Introduction/ Knowledge on diabetic foot condition | 1. Can you tell me what do you understand about your diabetic foot condition? Are you aware of what’s going on with your foot? 2. Do you know what are some complications with diabetic foot? 3. What are some factors that you know of that may put diabetic patients at risk for amputation? 4. How do you think your diabetic foot ulceration came about? Do you know what caused it? 5. How long did it take for you to get to see a doctor with your foot ulcer? 6. How long have you had your ulcer for? 7. What kind of shoes are you wearing at the moment? 8. Do you know what footwear options are there available for you in terms of offloading (i.e. diabetic shoes, wound sandals) 9. Do you know what is the best offloading option for a diabetic foot ulcer? 10. Have you been offered the total contact cast before? 11. Do you know what total contact casting is and how does it work? (To show picture of total contact cast if they are unsure how it looks like) |
| Perceptions of barriers to TCC | 12.  So.. I understand that you have been offered the cast before but you declined. What are some reasons why you would reject the idea of doing total contact casting? What do you think are some barriers to total contact casting?  (If need probing):   - intervention related: restriction of mobility and ADLs, unable to work, discomfort, sweating/itching, having to come in frequently for dressing change - patient related: appearance, psychological fear of being “trapped”, costly - practitioner related: unable to trust the clinician to do a good job, clinical expertise - wound related: fear of infection, unable to open wound for review   13.  Are there any factors why you would choose other offloading modalities compared to total contact casting even though you know it is the best treatment option? |
| Perceptions of enablers to TCC | 14.  As you have done total contact casting before,  what are some reasons why you can accept the idea of doing total contact casting?  (If need probing):  ·  gold standard offloading  ·  trust in clinicians  ·  alternative modalities did not work |
| Lived experience of TCC (for those who used TCC) | 15. How was your experience with total contact casting? 16. What was your first impression of doing the cast? 17. How long do you remember wearing the cast for? 18. How would you describe the feeling of wearing the cast? 19. What do you think are some advantages to using it? 20. What do you think are some disadvantages to using it? 21. Would you be open to wearing it again if you had the choice? 22. Would you be open to wearing it again if your wound requires it? 23. (If you have tried the cast and stopped using it) – what made you stopped using the cast? 24. What would make it easier for you to wear the cast? E.g. modifications to cast, support system from family etc 25. Would you recommend people to be on the cast if their wound requires it? |
| Roundup | Thank you so much for contributing your time to us today.  Is there anything else about your experience or views of total contact casting that I should know of? |

**Supplementary Information S3: Additional Quotes**

| **Themes** | **Sub- Themes** | **Quotes** |
| --- | --- | --- |
| Patient related barriers | Impact on mobility and daily functioning | “The sleeping is only the direction… it’s not easy also… you can’t turn uh too often. You have to lie flat on the bed.” (P02)  “The thought of being in a cast and having difficulty to shower, toilet and go out just to the shops... I think it's very inconvenient for me lah.” (P08)  “I see like four years I've been coming here to do the treatment. But I think if I wear the boot, I don't think so it will be taking me four years. But it's just that like I said, financial and inconvenience.” (P05)  “Like when I sit on the floor and get up. It’s a bit troublesome. Like I need something to… like to press or help me to get up...” (P01)  “The thing cover down there I cannot clean up that part that been covered and I feel very itchy, then a lot of germs around there that can’t be reached to be clean.” (P04)  “Because it's heavy, sometimes you cannot go left, you cannot go right, it's difficult. The whole foot cannot go down... Then when you used to go toilet to do your showering that's a difficulty lah.” (P11) |
|  | Lack of social support | “When you need to take a shower and you're in a cast, what if the patients are elderly or they are alone at home? ...And even with family support, how are they going to help you if you shower? The cast will definitely get wet. No matter what you do... you can buy something expensive online and you cover it up, but it will still get wet. It is not 100% waterproof.” (P08) |
| Intervention related barriers | Reduced means to monitor wound | “I won't be able to see if the wound is healing. And you cannot scratch and itch if your foot is itchy…” (P08)  “Yeah, also don't know what's happened in between the wounds, like suddenly got blister… so I have to come back immediately to the hospital, so not like as and when, you open.. and see is it getting better or getting worsen...” (P03)  “Very stressed. My bandage very easy. I take scissors I cut. Like that one, difficult.” (P06) |
|  | Changes in gait patterns | “Walking will be a different method already. So when the patient do walking, he or she have to be more careful.” (P02)  “To adapt… definitely not easy to control. You have to force yourself to control every single step...” (P02)  “Oh, yeah, like the movement also like sure be affected... I mean just imagine you're walking in the traffic light, like you want to move fast... also quite difficult lah.” (P03)  “Go jalan-jalan all, don't go. Take bus also difficult. Climb up, must see. Cannot, cannot bend anything.. Wait the cast, crack” (P07) |
|  | Cast therapy expectations | “This is a permanent thing, maybe you will put like u not like say those dressing, you can take out anytime, change or you want to put, you can. But this one if you put, I mean one month or two months or a few weeks...” (P03) |
|  | Cast complications | “Cast also can drop. Ya, sometimes the one ah, feel like high, sometimes low, flat, sometimes come big.” (P07) |
| Patient related enablers | Trust in clinicians’ expertise | “To me, every clinician, every podiatrist is a good one to me lah... I think they are all professional enough to be able to do a good job...” (P08) |
|  | Benefits of TCC | “There’s a reason why if I need to put on the cast I’ll have to go thru... Cuz I went through a lot of experience and I do understand everything for me is helping me... the cast will make sure, able to offload a lot of pressure when the wound part start to recover.” (P02)  “But I think for the sake of to fix my bones in line, I think I have to bear with that for the uncomfortable feeling.” (P10) |
